# Some aspects of the validity of the Montreal Cognitive Assessment (MoCA) for evaluating cognitive impairment in Brazilian patients with Parkinson's disease

**DOI:** 10.1590/s1980-5764-2016dn1004013

**Published:** 2016

**Authors:** Vitor Tumas, Vanderci Borges, Henrique Ballalai-Ferraz, Cyrus P. Zabetian, Ignácio F. Mata, Manuelina M.C. Brito, Maria Paula Foss, Nathalia Novaretti, Bruno Lopes Santos-Lobato

**Affiliations:** 1 Universidade de Ribeirão Preto, Faculdade de Medicina, Departamento de Neurologia, Ribeirão Preto, SP, Brazil.; 2 Universidade Federal de São Paulo, Escola Paulista de Medicina, Departamento de Neurologia , São Paulo, SP, Brazil.; 3 University of Washington School of Medicine, Seattle, Washington – Department of Neurology – Seattle, Washington, United States.; 4 Universidade de São Paulo Faculdade de Medicina de Ribeirão Preto - Neuroscience and Behavior, Ribeirão Preto, SP, Brazil.

**Keywords:** MoCA, validity, Parkinson's disease, mild cognitive impairment, dementia, cognitive assessment

## Abstract

**Background:**

The Montreal Cognitive Assessment (MoCA) is a short global cognitive scale, and some studies suggest it is useful for evaluating cognition in patients with Parkinson's disease (PD). However, its accuracy has been questioned in studies involving patients with low education.

**Objective:**

We sought to assess whether some of the MoCA subtests contribute to the low accuracy of the test.

**Methods:**

We performed a cross-sectional retrospective analysis of clinical data in a cohort of 71 patients with PD, most with less than 8 years of education. Patients were examined using the MDS-UPDRS, Hoehn and Yahr and the MoCA. The data were analyzed using mainly descriptive statistics.

**Results:**

We analyzed the data of 66 patients that were not demented according to the clinical evaluation and classified them using the proposed cut-off MoCA scores for diagnosis of MCI and dementia. Thirteen patients (19.7%) were classified as having normal cognition, 24 (36.3%) MCI and 29 (43.9%) dementia. Patients with dementia had longer disease duration (p=0.016) and lower education (p=0.0001). Total MoCA scores had a an almost normal distribution with a wide range of scores and only one maximum score. Performance on the MoCA was highly correlated with education (correlation coefficient=0.66, p=0.0001). At least five of the 10 MoCA subtests showed significant floor effects.

**Conclusion:**

We believe that some of the MoCA subtests may be too difficult to be completed by PD patients with low educational level, thus contributing to the test's poor diagnostic accuracy.

## Abstract (pt)

**Embasamento:**

A MoCA é uma escala cognitiva global breve, e alguns estudos sugerem que ela seria útil para avaliar a cognição em pacientes com doença de Parkinson (DP). No entanto, sua acurácia foi questionada em estudos em pacientes com baixa escolaridade.

**Objetivo:**

Pretendeu-se avaliar se alguns dos subtestes da MoCA poderiam contribuir para a baixa precisão do teste.

**Métodos:**

Foi realizada uma análise transversal e retrospectiva de dados clínicos de uma coorte de 71 pacientes com DP, a maioria com menos de 8 anos de escolaridade. Os pacientes foram examinados usando o MDS-UPDRS, a Hoehn e Yahr e a MoCA. Os dados foram principalmente analisados pela estatística descritiva.

**Resultados:**

Foram analisados os dados de 66 pacientes que não foram diagnosticados com demência de acordo com a avaliação clínica. Eles foram em seguida classificados, usando as notas de corte MoCA propostos para diagnóstico de comprometimento cognitivo leve (CCL) e demência. Dessa forma, treze pacientes (19,7%) foram classificados como com a cognição normal, 24 pacientes (36,3%) com CCL e 29 pacientes (43,9%) como com demência. Os pacientes com demência tiveram maior duração da doença (p=0,016) e menor escolaridade (p=0,0001). A distribuição dos escores totais da MoCA apresentaram forma de distribuição normal com uma vasta gama de pontuações e apenas uma pontuação máxima. O desempenho no MoCA foi altamente correlacionado à escolaridade (coeficiente de correlação=0,66, p=0,0001). Pelo menos cinco dos 10 subtestes da MoCA mostraram efeitos piso significativos.

**Conclusão:**

Alguns dos subtestes MoCA podem ser muito difíceis de completar por pacientes com DP com baixa escolaridade, contribuindo assim para a baixa precisão diagnóstica do teste.

## INTRODUCTION

The Montreal Cognitive Assessment (MoCA) is a short global cognitive scale designed to detect subjects with mild cognitive impairment (MCI).^1^ The aim of the developers was to provide a practical tool to uncover subtle cognitive changes that were frequently undetected by similar tests such as the Mini-Mental State Examination (MMSE). The MoCA test showed good internal consistency, test-retest reliability and content validity, and the original authors observed that the MoCA was considerably more sensitive than the MMSE for detecting MCI.^1^ They also showed that the scale was accurate for diagnosing mild Alzheimer's dementia.^1^

Further studies confirmed that the MoCA was better than the MMSE for detecting cognitive impairment, and that this was particularly evident in patients with Parkinson's disease (PD).^2-4^ Cognitive impairment is a common clinical problem in the course of PD, and is associated with decreased quality of life, increased caregiver burden, higher mortality rates, higher risk for institutionalization, and greater treatment costs.^5^ Thus, cognitive assessment is a key factor in evaluating patients with PD in research or clinical settings.

The MoCA test was translated and adapted for use in Brazil, and some studies have suggested that the MoCA is a valid and reliable tool for screening MCI in the Brazilian population.^6,7^ However, a study conducted by our group indicated that the scale may not be as good for diagnosing MCI in Brazilian patients with PD.^8^ We suggested that the lower educational level of our patients was the main reason for the poor performance on the MoCA, hence affecting the detection of MCI in the sample. The impact of education was evident in the original article, showing that patients with 12 years of education or less tended to have worse performance on the MoCA.^1^ Consequently, the authors proposed that to correct for education effects, 1 point should be added to total MoCA score (if <30) for participants with 12 years of education or less . Other studies confirmed that MoCA scores were strongly dependent on years of education.^9^

The MoCA is a 30-point test administered in 10-15 minutes, and through 10 subtests it can evaluate visuospatial/executive abilities (0-5 points), naming (0-3 points), working memory (0-2 points), attention (0-1 point), concentration/calculation (0-3 points), repetition (0-2 points), verbal fluency (0-1 point), abstraction (0-2 points), short-term memory (0-5 points) and orientation to time and place (6 points). One study conducted in illiterate or low-educated African subjects revealed that some of the MoCA subtests appeared to be difficult for these individuals, and the authors suggested that some adjustments in the test could improve its accuracy for use in this population.^10^ When the test given is too easy and all the subjects score very highly, a ceiling effect is observed. Conversely, when the task is too difficult, most subjects score very low and a floor effect is seen. The presence of significant ceiling and floor effects can influence the sensitivity and responsiveness of measuring instruments. The total MoCA score seems to be less prone to ceiling effects than the MMSE, explaining its better performance for screening MCI and mild dementia. However, if some of the MoCA subtests were too difficult for low education subjects, very low scores would be observed even for those without cognitive impairment. A possible floor effect of some of the subtests in this population could explain the low sensitivity of the MoCA, and some adaptation of the test would therefore be necessary to improve its performance for detecting MCI and mild dementia in illiterate and low-educated individuals.

The objective of this study was to retrospectively analyze performance on the MoCA test of a sample of Brazilian PD patients, and to assess whether some of the subtests might contribute to the low accuracy of the test in the detection of MCI among these patients.

## METHODS

In this study, a cross-sectional retrospective analysis was performed using clinical data obtained from a cohort study investigating the genetic characteristics of Latin American patients with PD. The LARGE-PD (Latin American Research consortium on the GEnetics of Parkinson's Disease) study started in 2005 as a multicenter study involving several participating centers in Latin America under the coordination of Dr. Ignacio Fernandez Mata, and Dr. Cyrus Zabetian at the University of Washington. Two Brazilian centers have since been collecting clinical data and DNA samples for 430 participants, comprising 201 patients with PD and 229 control subjects. The data analyzed in this study were obtained from patients from these 2 centers, the Ribeirão Preto Medical School (USP) and the Universidade Federal of São Paulo (UNIFESP).

Patients were included if diagnosed with PD according to the United Kingdom Brain Bank diagnostic criteria.^11^ They were invited to participate if they were older than 18 years and agreed to take part in the study.

Patients were examined by a neurologist who evaluated them using the MDS-UPDRS, the Hoehn and Yahr scale (H&Y) and the Brazilian version of the MoCA. Other clinical and epidemiological data were also collected. The same neurologist applied the MoCA and determined the degree of cognitive impairment according to the specific item of the MDS-UPDRS based on his professional opinion. He did not specifically take into account patient score on the MoCA test to rate the cognitive item of the UPDRS. Patients with motor fluctuations were all evaluated in the "on-state". The local research ethics committee approved the study and all participants or their relatives provided signed informed consent to participate.

The data were analyzed mainly using descriptive statistics. The descriptive frequency of ceiling and floor scores of the MoCA subtests were the main focus of the analysis. For description of ceiling and floor scores, the number of maximum and minimum scores obtained by the patients on the different MoCA subtests were evaluated, respectively. A significant floor effect was defined when ≥20% of the patients obtained minimum scores on the subtest. The data were also evaluated using the Chi-square test and non-parametric statistics: the Mann-Whitney test and the Kruskall-Wallis test with Dunn's post-hoc test.

We calculated Spearman's correlation coefficients between the scores on the MoCA and age, schooling and disease duration. To determine the data distribution pattern, the Shapiro-Wilk test, visual inspection of the Q-Q plots and measures of skewness and kurtosis were used. The statistical analysis was performed using the SPSS 19 and the level of statistical significance was defined as p<0.05.

## RESULTS

Initially, data of 71 patients with PD from the LARGE-PD study were included for analysis. The data of the other 130 patients were not included for analysis because they were incomplete. More specifically, the scores for the MoCA subtests had not been registered in the clinical protocol (only total score) for most of these subjects. From this initial sample, cases with possible diagnosis of dementia according to the UPDRS item 1: cognitive impairment (score >1) were excluded. The data for the remaining 66 patients that scored 0 or 1 on the cognitive impairment item of the MDS-UPDRS according to the examiner's opinion were then selected for analysis. The data for 5 patients was thus excluded from the analysis.^12^ This procedure was performed to establish a cohort that would be most likely composed of PD patients with MCI or normal cognition. The demographic and clinical features of these 66 patients with PD included in the analysis are given in Table 1.

**Table 1:** Demographic and clinical features of the sample of patients with PD.

Number of patients		66
Gender (male/female)		41/25
Age (years)		61 (39-83)*
Education (years)	0-4	26 (39.4%)
	5-8	17 (25.7%)
	>8	23 (34.9%)
Disease duration (years)		8 (1-31)*
Hoehn and Yahr score	1	3
	2	48
	3	13
	4	2
	5	0

* median (min-max).

In this sample of data for the 66 patients, and adopting the proposed cut-off MoCA scores for diagnosis of MCI and PDD in the sample, 13 patients (19.7%) were classified as having normal cognition, 24 (36.3%) MCI and 29 (43.9%) as having PDD according to performance on the MoCA. The cut-off scores used for this classification were<21 for diagnosis of dementia and 21-25 for diagnosis of MCI. The non-parametric Mann-Whitney test showed that patients with PDD diagnosed according to the total MoCA score had longer disease duration (p=0.016) and lower education (p=0.0001) than the other patients.

**Figure 1:**
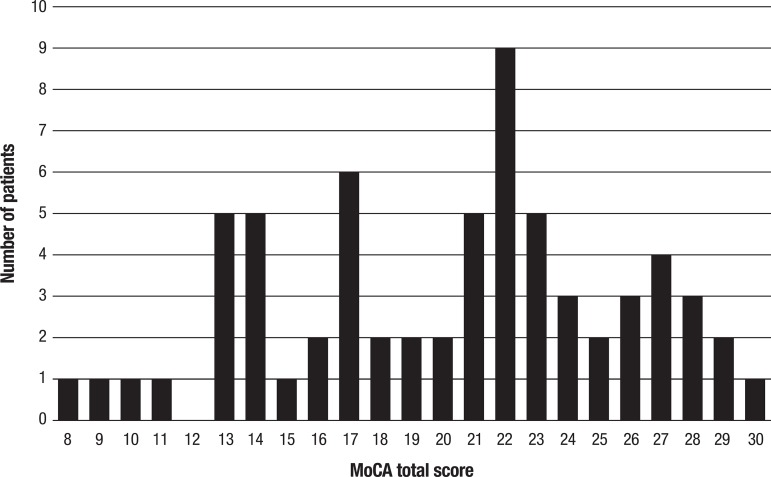
Graph showing distribution of total MoCA scores in the sample of 66 Brazilian patients with PD. Total scores were approximately normally distributed with a wide range of scores and only one patient obtaining the maximum score and none obtaining the minimum score.

Figure 1 depicts the performance of the patients on the MoCA test showing the distribution of total MoCA scores across subjects. The Shapiro-Wilk test (p=0.101) and visual inspection of the histogram and Q-Q plots showed that the test scores were approximately normally distributed, with skewness of –0.253 (SE=0.295), and kurtosis of –0.75 (SE=0.582). There was a wide range of scores from 8 to 30 points, with only one patient obtaining the maximum score and none obtaining the minimum score.

A significant correlation was observed between total MoCA scores and education (cc=0.66, p=0.0001) and duration of the disease (cc= –0.31, p=0.011) but not with age (cc= –0.23, p=0.059).

**Figure 2:**
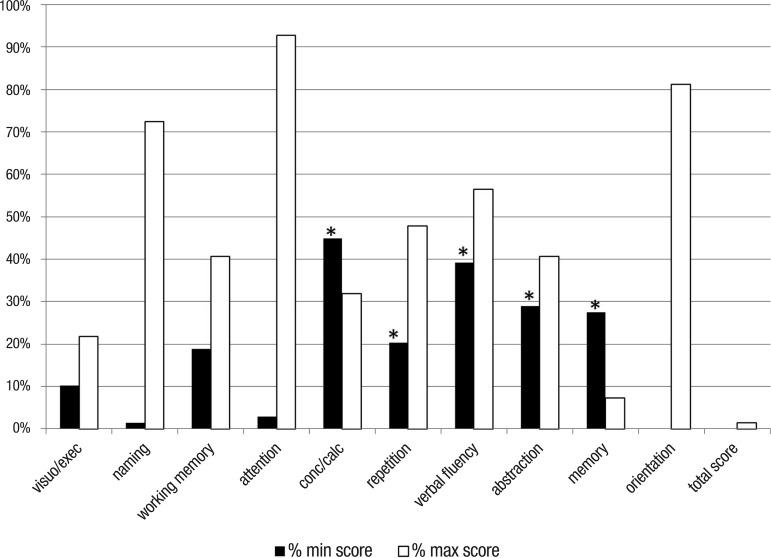
Graph showing percentage of minimum and maximum scores on the MoCA subtests: visuospatial/executive abilities (0-5 points), naming (0-3 points), working memory (0-2 points), attention (0-1 point), concentration/calculation (0-3 points), repetition (0-2 points), verbal fluency (0-1 point), abstraction (0-2 points), short-term memory (0-5 points) and orientation to time and place (6 points). There was a significant floor effect in 5/10 of the MoCA subtests (≥20%). *≥20% of patients obtained minimum scores on subtest.

Figure 2 shows the percentage of maximum and minimum scores observed on each MoCA subtest and for the total MoCA score. A significant floor effect was observed in 5 out of the 10 MoCA subtests: concentration/calculation (serial 7), language repetition, verbal fluency, abstraction and memory. A significant ceiling effect was found on the other 3 subtests: naming, attention and orientation; and a low incidence of minimum scores was observed.

## DISCUSSION

Many studies have considered MoCA a much better tool than the MMSE for evaluating cognition in patients with PD.^3,4,13-15^ However, there is evidence from some studies that when the test was used in low or mid-educated subjects its diagnostic accuracy decreases.^8-10^ On the other hand, some studies showed no such interference of years of education on patient performance in the MoCA test.^16,17^

In our study, we retrospectively evaluated the data from a sample of PD patients without a dementia diagnosis based upon the MDS-UPDRS cognitive item. We observed that the application of the proposed MoCA cut-off scores for screening cognitive impairment rated 29 of the patients as having PDD. Even considering the fact that the examiner was not primarily focused on the diagnosis of the cognitive status of patients and that some may have been misdiagnosed, the proportion of cases classified as PDD by the MoCA total cut-off score was very high (43.9%). These patients were older and lower educated compared to the others in the sample. The association between older age and lower education with the diagnosis of PDD is relatively frequent, and there is substantial evidence to suggest that they are well-defined risk factors for the development of dementia.^5^ However, we could speculate that our patients with low MoCA scores had worse performance on the test because of their lower educational level and not just because they had significant cognitive impairment. Most patients included in this study had less than 8 years of education (65%), and as cited previously, performance in the MoCA is highly dependent on years of education as observed in our study.^1,8^

When we analyzed the distribution of the total MoCA scores in our patients, we found that it assumed an almost normal pattern, with a wide range of scores and no significant floor or ceiling effects as previously reported.^3^ However, analyzing the different subtests, we found that at least five had a significant floor effect, suggesting that they were too difficult to complete by our patients, and maybe because of this, many subjects scored zero. The concentration/calculation subtest requires the subject to perform a serial 7 subtraction (100-7), and patients must err on all 5 subtractions to obtain a zero score. The repetition subtest requires the repetition of two unusual and relatively long phrases, and a zero score indicates the subject could not repeat any of them. The lexical verbal fluency test requires the generation of at least 11 words in one minute to score one on the task. The abstraction subtest evaluates the ability of the subject to recognize the categorical similarity between two objects twice, and a zero score indicates that they were unable to recognize any of them. Finally, the memory subtest requires the subject to evoke at least one word out of five to score above zero. We could argue that the first four subtests may actually represent difficult tasks for our patients. However, the low performance by the same case on the memory test cannot be easily explained.

However, our patients showed very good performance on the naming, attention and orientation subtests, and interestingly they also performed very well on the visuospatial/executive function subtest. This is surprising, since performance on both these subtests seems to be affected by lower education as reported by other studies.^9,10^

Our study has many limitations. The main one is that it was a retrospective analysis of the performance on the MoCA and a comparative gold standard evaluation of the cognitive status of our patients was not performed. Indeed, we cannot definitively state that the patients included in the analysis did not have a dementia diagnosis. Despite these limitations, our findings may help understand the apparent lower diagnostic accuracy of the MoCA test for diagnosing cognitive status in low-educated patients. Significant floor effects of some of the MoCA subtests could compromise the accuracy of the test, and it is possible that some simple adaptations in the application approach of the MoCA could improve its performance. For example, the substitution of the lexical verbal fluency test by a semantic verbal fluency test, as well as adaptation of the scoring on the subtest for our population. The sentences in the repetition subtest could be simplified while the categories of similarities could be made easier. It would be more complicated to simplify the calculation and memory test. Perhaps, emphasizing the instruction that patients should memorize the words because they will be asked to evoke them later, could help improve their performance. Another alternative would be to facilitate the learning of the 5 words by linking them with their semantic characteristics, as done in other memory tests.

These are all exploratory general observations that should be reproduced by other larger, well-designed studies.

In conclusion, it is possible that some of the MoCA subtests are too difficult to be completed by patients with lower education thereby contributing to the test's poor diagnostic accuracy. It seems that more consistent normative data is needed to use the MoCA test as a reliable tool for diagnosing cognitive impairment in low-educated patients with PD.
